# Vertebral Fracture in a Short Period Following Posterior Decompression and Fixation for Degenerative Lumbar Spondylolisthesis

**DOI:** 10.7759/cureus.49137

**Published:** 2023-11-20

**Authors:** Yoshinori Maki, Kenji Fukaya

**Affiliations:** 1 Neurosurgery, Hikone Chuo Hospital, Hikone, JPN; 2 Neurosurgery, Ayabe Renaiss Hospital, Ayabe, JPN

**Keywords:** complication, vertebral fracture, pedicle screw, posterior lumbar interbody fusion, degenerative lumbar spondylolisthesis

## Abstract

Posterior decompression and fixation are established therapeutic modalities for degenerative lumbar spondylolisthesis (DLS). Postoperative complications associated with these procedures may require supplementary interventions, potentially resulting in subsequent vertebral fractures. However, vertebral fractures that occur within a short period after posterior decompression and fixation for DLS are rare. An 80-year-old woman presented with right leg pain and ambulatory difficulties attributed to DLS. The patient was administered medications, including prednisolone, for managing diabetes mellitus and rheumatoid arthritis. Subsequently, the patient underwent posterior decompression from L3 to S1, coupled with fixation extending from L4 to S1 using percutaneous pedicle screws. The symptoms disappeared, and the patient was discharged two weeks after the surgery. However, two months after the surgery, the patient visited our outpatient clinic, complaining of sudden backache and motor weakness in the bilateral lower extremities. A vertebral fracture of L4 was identified on computed tomography (CT). Long-level fusion from Th10 to the iliac bone was performed to correct the thoracic-lumbar deformity. Following rehabilitation therapy after the second surgery, the patient was discharged on day 45 post-surgery. As observed in this case, vertebral fractures following posterior decompression and fixation surgery for DLS can occur within a relatively short period. Neurosurgeons should be aware of these rare complications.

## Introduction

Degenerative lumbar spondylolisthesis (DLS) results from the degeneration of facet joints and loosened ligaments. These conditions can lead to the forward slipping of the vertebral body onto the one caudal [1]. Degenerative lumbar spondylolisthesis typically occurs after the age of 50, with a higher incidence in women than in men. Patients with DLS can suffer from lower back and leg pain related to the lumbar canal or foraminal stenosis [2]. Extensive research supports the favorable outcomes of surgical management for DLS over conservative management [3,4]. Specifically, posterior decompression and fusion surgery for DLS can result in superior postoperative outcomes than using posterior decompression alone [5].

However, in clinical practice, patients with DLS require reoperation owing to hemorrhagic events, dural tears, and wound-related issues, such as dehiscence and infection, recurrent stenosis, and progressive spondylolisthesis [6-8].

Although subsequent vertebral fractures occurring at the adjacent, instrumented, or remote levels after posterior fixation for DLS can also be a complication requiring additional surgical procedures, this manifestation is considered rare [7,8]. Furthermore, within the existing literature, there is a paucity of cases reporting vertebral body fractures that manifest in a relatively short period following posterior decompression and fixation surgery for DLS, resulting in thoracic-lumbar spinal deformity and the need for thoracic-lumbar long fixation surgery.

Herein, we describe a case of this rare complication, emphasizing the need for close follow-up after fixation and decompression surgery for DLS.

## Case presentation

**Figure 1:**
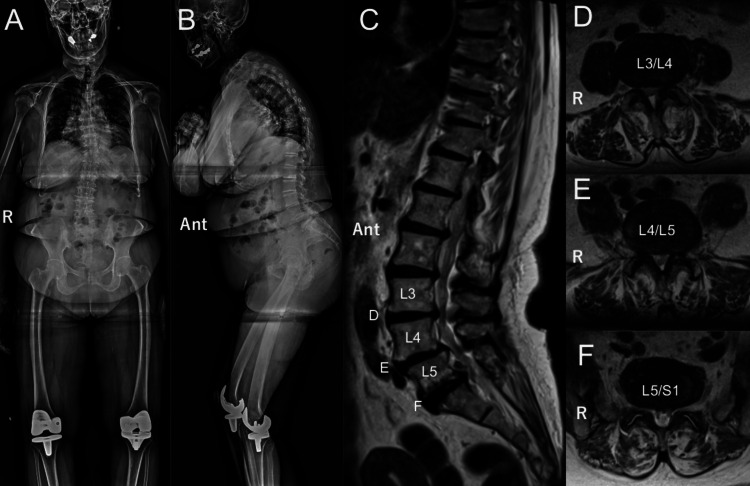
Preoperative radiographic images A: anterior-posterior projection; B: lateral projection; radiographic images display the spinal deformity resulting from lumbar spondylolisthesis; (C) A sagittal magnetic resonance image (MRI) reveals lumbar canal stenosis from L3 to S1 and spondylolisthesis at the L4/5 level; (D-F) The axial MRI images indicate lumbar canal stenosis at distinct levels (d: L3/4, e: L4/5, and f: L5/S1) (Ant: anterior, R: right).

An 80-year-old woman presented with pain in the right lower extremity and ambulation difficulty that had persisted for over four months. She was under medication for diabetes mellitus and received prednisolone (5 mg) for rheumatoid arthritis. Radiological images revealed lumbar canal stenosis from L3 to S1 and spondylolisthesis at L4/5 (Figure 1).

**Figure 2:**
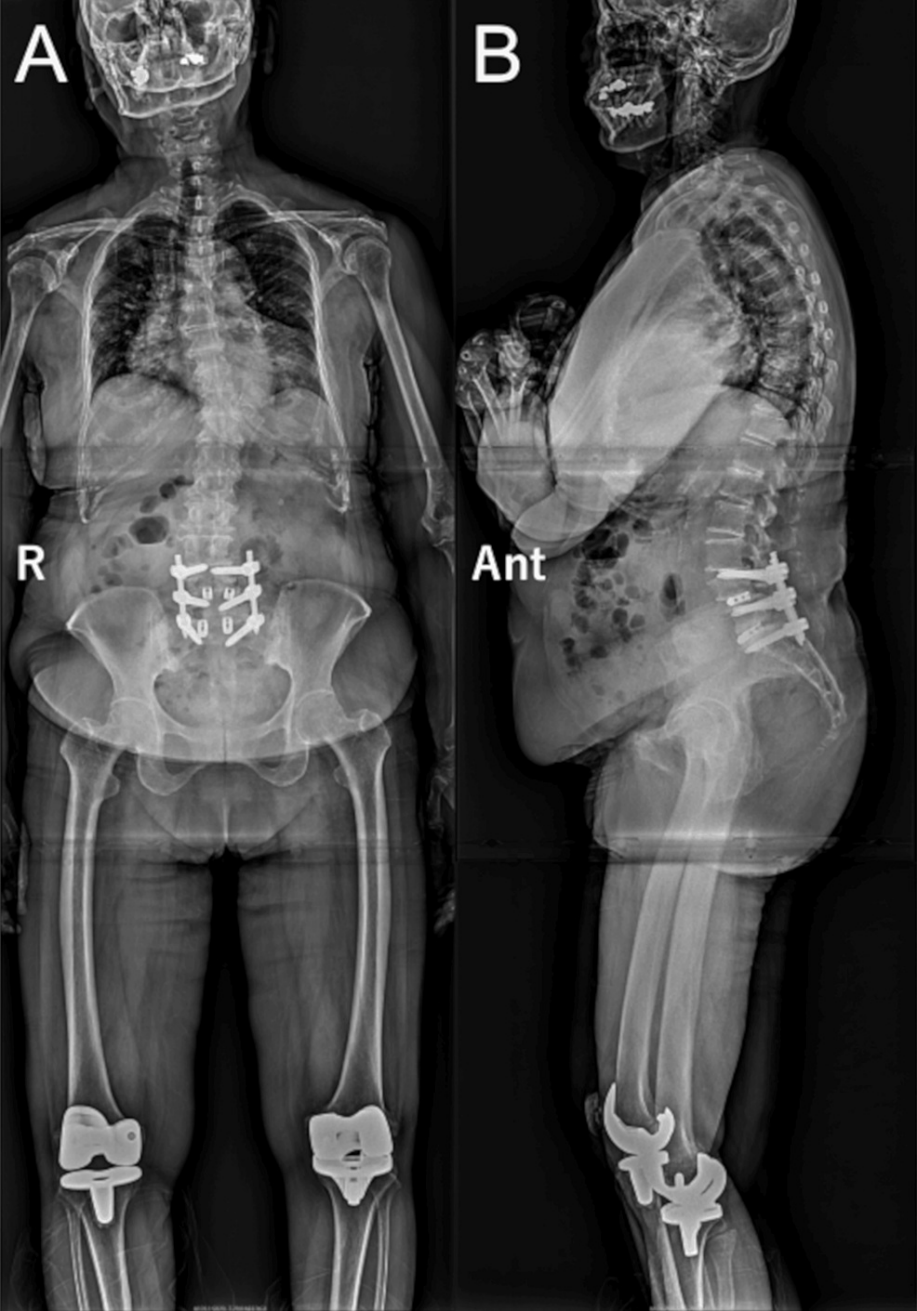
Radiographic images after the first operation A: anterior-posterior projection; B: lateral projection; posterior lumbar intervertebral fusion from L4 to S1 is performed (Ant: anterior, r: right).

Given that these findings correlated with the symptoms of the patient, we planned surgical treatment. At first, we thought that decompression surgery from L3 to L5 seemed enough. However, as we did not completely exclude that the symptoms of the patient could have been related to the radiculopathy, we performed posterior lumbar decompression from L3/4 to L5/S1 and posterior lumbar intervertebral fusion of L4/5 and L5/S1 using percutaneous pedicle screws. The bilateral pedicle screws were inserted to avoid interference between them in the same vertebral body (i.e., a right pedicle screw was tilted slightly to the cranial direction while a left one was tilted slightly to the caudal direction, and vice versa) (Figure 2).

**Figure 3:**
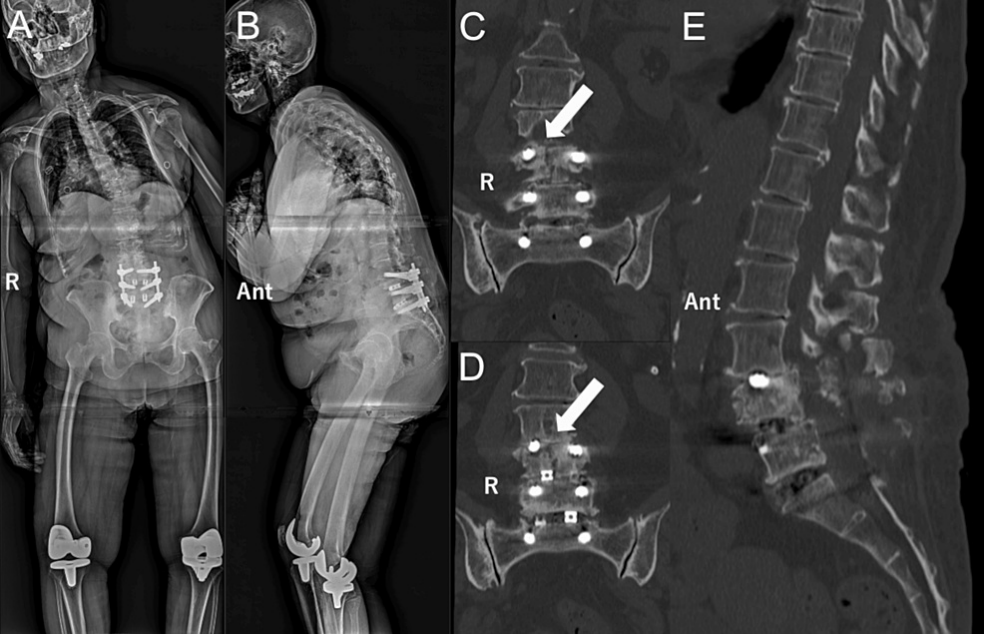
Radiographic images after the vertebral fracture of L4 A: anterior-posterior projection; B: lateral projection; C, D: coronal computed tomography images; E: sagittal computed tomography image. Because of the vertebral fracture of L4 (white arrows), the spinal deformity was aggravated (Ant: anterior, R: right).

Symptoms were relieved, and the patient was discharged from the hospital two weeks after surgery. However, two months later, the patient presented to our outpatient clinic complaining of sudden backache as well as bilateral toe paralysis. Manual muscle testing revealed bilateral motor weakness. Consequently, the patient was unable to ambulate independently. Radiography and computed tomography (CT) images revealed a vertebral body fracture of the L4 vertebra and a thoracic-lumbar spinal deformity (Figure 3).

**Figure 4:**
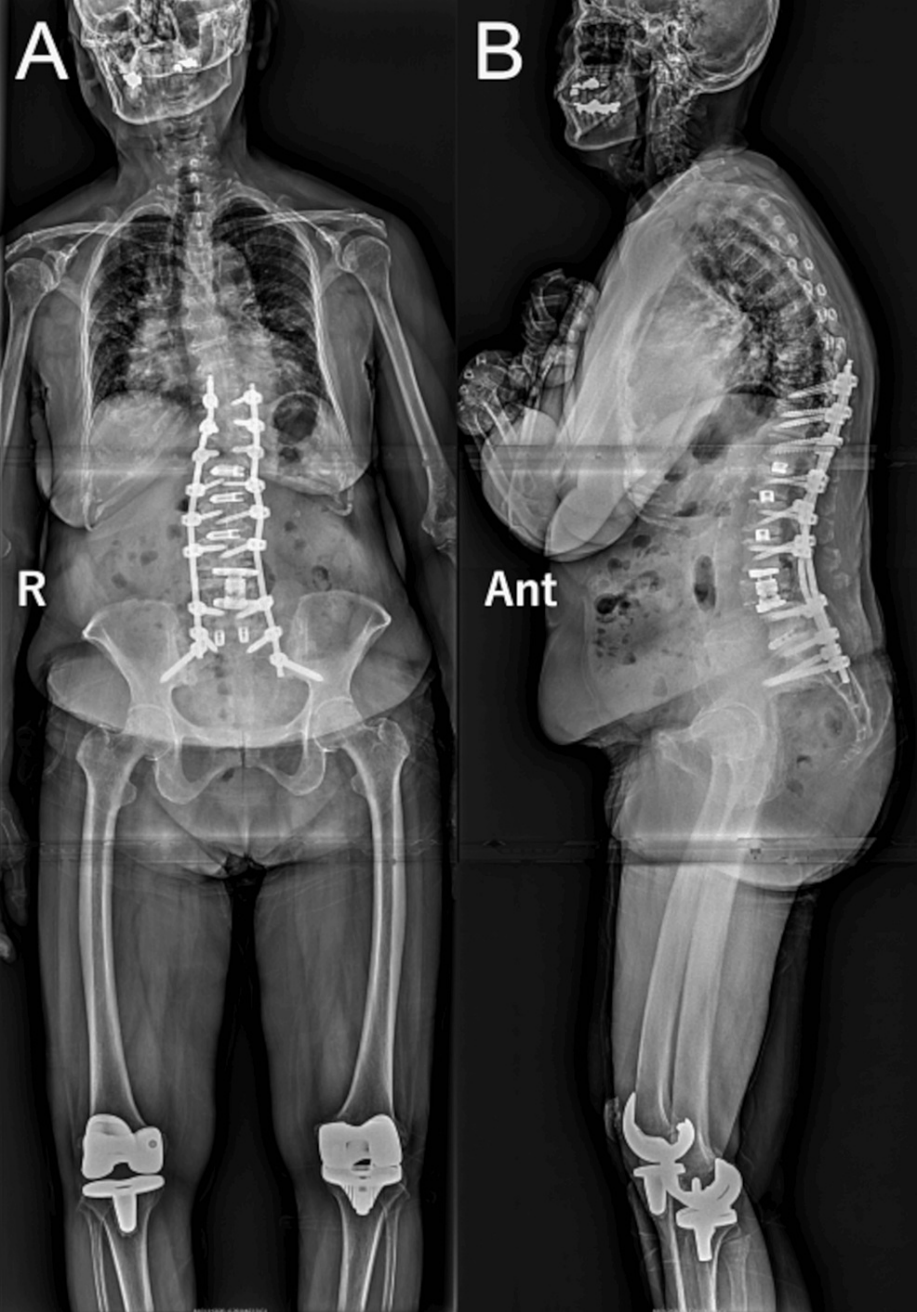
Radiographic images four months after the second operation A: anterior-posterior projection; B: lateral projection. The spinal deformity was corrected using posterior thoracic-lumbar-iliac long fusion (Ant: anterior, r: right).

We considered that the thoracic-lumbar spinal deformity could have resulted from a vertebral body fracture of the L4 vertebra. Although an additional fixation surgery of two or three levels above seemed enough, we thought that there could have been a risk of another fracture or loosening of the placed screws. These conditions seemed to lead to another repair surgery. Therefore, to resolve the pain induced by the fracture and deformity in a single session, oblique lumbar intervertebral fixation from Th12/L1 to L2/3, posterior fixation from Th10 to the ilium using percutaneous pedicle screws, and L4 vertebral body replacement were performed. The postoperative clinical course of the patient was uneventful. Two days after the second surgery, the patient walked 20 meters with the support of a walker. Subsequent rehabilitation therapy resulted in improved activities of daily living. The patient was discharged 45 days following the second surgery. Radiographic assessments four months after the second surgery confirmed the resolution of the spinal deformity (Figure 4, Table 1).

**Table 1:** Chronological changes in the parameters related to spinal deformities on radiographic images PT: pelvic tilt; SS: sacral slope; PI: pelvic incidence; LL: lumbar lordosis; TK: thoracic kyphosis; TPA: thoracic 10 pelvic angle; SVA: sagittal vertical axis

Parameters	Before the first surgery	One month after the first surgery	Before the second surgery (after the L4 vertebral fracture)	Four months after the second surgery
Cobb angle	20.2°	19.8°	18.0°	16.8°
Coronal balance	3.9 mm	14.1 mm	124.6 mm	35.8 mm
PT	26.1°	31.0°	32.2°	34.0°
SS	47.3°	39.0°	36.8°	33.3°
PI	73.4°	70.0°	69.0°	67.3°
LL	47.0°	54.6°	29.9°	44.6°
PI-LL	26.4°	15.4°	39.1°	22.7°
TK	51.6°	53.0°	57.9°	55.4°
TPA	37.7°	28.9°	48.5°	36.5°
SVA	168.5 mm	58.0 mm	211.8 mm	108.7 mm

## Discussion

We present the case of DLS in a patient who underwent posterior decompression and fixation. In our case, a vertebral fracture occurred as a rare postoperative complication, manifesting two months after posterior decompression and fixation. Consequently, additional long-level fixation extending from Th10 to the iliac spine was required to address the spinal deformity resulting from the vertebral fracture. To the best of our knowledge, vertebral fractures occurring shortly after posterior decompression and fixation for DLS have not been previously documented.

As complications following posterior decompression and fixation for DLS, vertebral fractures are considered rare, even though these fractures can occur at adjacent, remote, or instrumented levels [7,8]. In our case, the vertebral fracture was observed at the cranial instrumented level, L4. Previously, Toyone et al. analyzed subsequent vertebral fractures following posterior fixation surgery for degenerative lumbar disease. One hundred patients (aged ≥55 years) were recruited for their study. In their study, 12 patients who underwent posterior fixation from L4 to S1, similar to that administered to our patient, were included [8]. Toyone et al. concluded that subsequent vertebral fractures could occur in postmenopausal women within two years of surgery [8]. Nakahara et al. also reported that the risk of subsequent vertebral fractures after posterior lumbar fixation was higher in patients older than 70 years old [7]. Besides age and sex, several risk factors, such as diabetes mellitus, osteoporosis, and posterior lumbar intervertebral fusion, can result in proximal vertebral fractures after posterior lumbar fusion [9-11]. Additionally, rheumatoid disease and oral steroid administration can cause postoperative fracture complications [12]. Our patient was 80 years old and had a history of diabetes mellitus and rheumatoid arthritis. An oral steroid was administered preoperatively, and the patient underwent posterior lumbar intervertebral fusion for DLS; these conditions may have led to subsequent vertebral fractures. Another possible factor resulting in the vertebral body fracture of L4 could have been that the L4 pedicle screws violated the L4 superior endplate and intervertebral disc of L3-4. When we insert the pedicle screws in our institute, we usually tilt a pedicle screw on one side slightly in the cranial direction and tilt another one on the other side in the caudal direction. We perform this surgical procedure to avoid interference between pedicle screws in the same vertebral body. In most cases, however, we rarely encounter a vertebral body fracture related to our fashion of placing pedicle screws, which was observed in our case. However, because of the vulnerability of the bone in this case, the violation of the L4 pedicle screws might have resulted in this complication.

To avoid a vertebral fracture after posterior fixation surgery, preoperative medication such as teriparatide seems indispensable to strengthen bone tissue. Posterior fixation surgery should have been performed after confirming the amelioration of the bone tissue of the patient. Because our surgical fashion of inserting pedicle screws also might have resulted in a vertebral body fracture, the trajectory of the L4 pedicle screws should have been corrected intraoperatively so as not to violate the upper endplate of the L4 vertebral body. Perhaps, as we considered at first, only decompression surgery without fixation could have been an option to avoid the sequela.

Given the potential for subsequent vertebral fractures after posterior decompression and fixation surgery for DLS to be overlooked during postoperative outpatient follow-up and the limited description of this postoperative complication [13,7], our case warrants dissemination among neurosurgeons.

## Conclusions

In conclusion, vertebral fractures can occur within a short period after posterior decompression and fixation for DLS. In our case, the L4 vertebral fracture occurred possibly because of the patient's age, sex, underlying disease, and medication. A routine surgical procedure could have also resulted in this complication. Preoperative medication and intraoperative modification of surgical fashion may prevent this unfavorable outcome.
